# L925I Mutation in the *Para*-Type Sodium Channel Is Associated with Pyrethroid Resistance in *Triatoma infestans* from the Gran Chaco Region

**DOI:** 10.1371/journal.pntd.0002659

**Published:** 2014-01-23

**Authors:** Natalia Capriotti, Gastón Mougabure-Cueto, Rolando Rivera-Pomar, Sheila Ons

**Affiliations:** 1 Laboratorio de Genética y Genómica Funcional. Centro Regional de Estudios Genómicos. Universidad Nacional de La Plata, Buenos Aires, Argentina; 2 Centro de Investigaciones de Plagas e Insecticidas (CIPEIN, CITEFA-CONICET), Buenos Aires, Argentina; 3 Departamento de Ciencias Básicas y Experimentales. Universidad Nacional del Noroeste de la Provincia de Buenos Aires, Pergamino, Argentina; Mahidol University, Thailand

## Abstract

**Background:**

Chagas' disease is an important public health concern in Latin America. Despite intensive vector control efforts using pyrethroid insecticides, the elimination of *Triatoma infestans* has failed in the Gran Chaco, an ecoregion that extends over Argentina, Paraguay, Bolivia and Brazil.

The voltage-gated sodium channel is the target site of pyrethroid insecticides. Point mutations in domain II region of the channel have been implicated in pyrethroid resistance of several insect species.

**Methods and Findings:**

In the present paper, we identify L925I, a new pyrethroid resistance-conferring mutation in *T. infestans*. This mutation has been found only in hemipterans. In *T. infestans*, L925I mutation occurs in a resistant population from the Gran Chaco region and is associated with inefficiency in the control campaigns. We also describe a method to detect L925I mutation in individuals from the field.

**Conclusions and Significance:**

The findings have important implications in the implementation of strategies for resistance management and in the rational design of campaigns for the control of Chagas' disease transmission.

## Author Summary

One of the principal goals of the Southern Cone Initiative (SCI), created in 1991, is to reduce the geographic range and infestation prevalence of triatomines, vectors of Chagas'disease. Despite its success in many areas, the elimination of *Triatoma infestans* has failed in the Gran Chaco ecoregion, even in areas subjected to intense vector control efforts with pyrethroid insecticides. Detecting the reasons for the persistence of *T. infestans* in the Gran Chaco has been recognized as a priority of the SCI, in its 15^th^ meeting held in Brasilia in 2006. The voltage -dependent sodium channel is the target protein of pyrethroid insecticides. In this work, we investigated possible mutations in the sodium channel gene in a highly resistant population from Gran Chaco. We found a point mutation that affects the structure of the channel and is associated with high resistance to pyrethroids. We also present a simple and low-cost method to detect this mutation in individuals from the field. Our results are important for monitoring the presence and spread of pyrethroid-resistant populations of *T. infestans*, a critical requirement in the implementation of strategies for resistance management and for the rational design of campaigns oriented to controlling the transmission of Chagas' disease.

## Introduction

Chagas' disease is an important but neglected tropical disease that affects 10 million people in Central and South America; the insect vectors *Triatoma infestans* and *Rhodnius prolixus* are responsible for most of the cases in the continent (http://www.who.int/mediacentre/factsheets/fs340/en/). Because there is still no vaccine or effective treatment for the chronic stage of the disease, vector control remains the only method to reduce the risk of transmission.

The Southern Cone Initiative (SCI), created in 1991 by the governments of Argentina, Bolivia, Brazil, Chile, Paraguay and Uruguay, has reduced the geographic range and infestation prevalence of triatomine vectors [1], [2]. However, in the Gran Chaco ecoregion, the elimination of *T. infestans* has failed, even in areas subjected to intense vector control efforts using pyrethroid insecticides; the reasons for this failure are poorly understood [2], [3]. Determining the reasons for the persistence of *T. infestans* in the Gran Chaco has been recognized as a priority of the SCI, in its 15^th^ meeting held in Brasilia in 2006 [3].

Resistance to pyrethroids in triatomines has been detected in South America since the 90s [4]–[6], and high resistance levels that correlated with field control failures have been detected in *T. infestans* on the border between Argentina and Bolivia since 2002. Different levels of resistance have been reported in several areas of Argentina, Bolivia and Paraguay [7]–[10]. In the Chaco province of Argentina, at the heart of the Gran Chaco, resistance to deltamethrin has been found in the neighbor localities of La Esperanza and El Malá (G. Mougabure-Cueto unpublished observations; [10]). When analyzed with the topical application assay, the El Malá population displays an RR_50_ to deltamethrin of 1031 [10], corresponding to an extremely resistant population. In Pampa del Indio, located 103 km away from this area, residual insect populations were observed after four pyrethroid sprayings [11]. These residual insects were successfully eliminated with the organophosphate Malathion (unpublished observations).

Pyrethroids exert their insecticidal action on the insect nervous system by modifying the normal function of voltage-gated sodium channels in the membranes of excitable cells. Knockdown resistance (*kdr*) is the reduction in the sensitivity to pyrethroids caused by point mutations in the sodium channel gene [12]. Recently, we demonstrated the presence of the mutation L1014F as a *kdr* mutation in *T. infestans* from a resistant population from Salta province, Argentina [13]. The resistance ratio (RR) of this population was measured as 35.7. However, different resistance profiles and huge differences in RR observed in resistant populations from the field [5]–[10] suggest the existence of other resistant-associated alleles.

Here we identify the *kdr* mutation L925I, in *T. Infestans* from a resistant population, from a locality called El Malá, at the heart of the Gran Chaco ecoregion. Unlike L1014F, a pyrethroid resistance mutation found in many insect species from different families, L925I mutation has been identified only in hemipterans thus far [14]–[16]. Electrophysiological characterization by *in vitro* expression in *Xenopus laevis* oocytes proved that L925I substitution significantly decreases pyrethroid potency [17]. We also describe a method to detect L925I mutation in individuals from field populations, based on a different pattern of digestion of a PCR fragment by a restriction endonuclease.

The information presented here is useful for the early detection of the presence and spread of resistant populations, a critical requirement in the implementation of strategies for resistance management. Early detection of insecticide resistance is essential for the rational design of campaigns, oriented to controlling the transmission of Chagas' disease in all its distribution range.

## Methods

### Ethics statement

The rearing and use of insects and pigeons performed in this study was carried out according to the World Health Organization protocol [18] and approved by the Ethic Committee of the Research Center of Pests and Pesticides (National Council of Scientific and Technical Research, CONICET, Buenos Aires, Argentina). The protocol used is in complete accordance with the recommendations established by the Directive 2010/63/EU of the European Parliament, related to the protection of animals used for scientific purposes.

### Insect sampling and rearing

Reference *T. infestans* insects were obtained from an insecticide-susceptible (S) strain maintained in the laboratory without any exposure to insecticides. Samples from field population of *T. infestans* were collected in November 2010 from a small locality called El Malá, in the Argentinean Gran Chaco region (S25′56.077″ W60′27.105″). Insects were collected from infested houses, in a region where vector control using pyrethroid insecticides was considered ineffective by the authorities of the Chagas Program of Chaco province. The chemical treatment in the study area in the last five years prior to the collection was conducted in a discontinuous manner and was performed by different effectors (national and provincial). Further generations of the field-collected insects were raised in the laboratory without exposure to pyrethroids. The insects used in this work belong to F2 generation from field-collected individuals. Both colonies were reared at the laboratory under controlled temperature (28±1°C), humidity (50%–70%) and photoperiod (12∶12 L∶D), and were fed on pigeons.

### Molecular analysis of Susceptible (S) and Resistant (R) insects

Genomic DNA was extracted from 15 insects from each colony -susceptible (S) and resistant (R)- with a commercial kit, following the manufacturer's instructions (Promega, Madison, USA). Fragments of the sodium channel gene were PCR amplified using pooled genomic DNA from S or R *T. infestans* as templates.

Seminested PCR reactions were performed. Seminested PCR augmented the yield and the specificity of the primary PCR. The primary reaction (30 µl) used primers Ti Fwd and Ti Rev1 (Table 1) and contained 20 ng of DNA, 0.2 mM dNTP, 0.2 µM of each primer, 1.5 mM MgCl_2_, 1 U Taq Platinum DNA polymerase (Invitrogen, São Paulo, Brazil) and was run through 30 cycles of 30 s at 95°C, 50 s at 48°C, and 50 s at 72°C. One µl of this PCR was used as a template for a secondary seminested PCR reaction with similar parameters, but using the primers Ti Fwd and Ti Rev2 (Table 1). Finally, 1 µl of this PCR was used as template for a tertiary seminested PCR reaction with similar parameters, but using the primers Ti Fwd and Ti Rev3 (Table 1). PCR fragments were cloned using the pGEM-T Easy Vector System (Promega, Madison, WI, USA) and sequenced by capillary electrophoresis at Macrogen (Seoul, Korea). Sequences from pools of individuals belonging to S or R strains were compared (13 clones/strain were analyzed) in order to determine the presence of resistance-conferring mutations.

**Table 1:** Sequence of the primers used in this study.

Primer name	5′-3′ Sequence
Ti Fwd	TGGCCAACATTGAATTTATTGATATC
Ti Rev 1	TGTTACGATTTGATGATAACCGGGATA
Ti Rev 2	TGAACCTTGTTTCCAGCTGG
Ti Rev 3	TTAACCCGAACAAGAATATA
Ti Rev 4	AATATATAAAGTACTTACAACA

### PCR and restriction endonuclease assay

Ti Fwd and Ti Rev4 primers (Table 1) were used to amplify a fragment of the sodium channel from S and R populations. Ti Fwd and TiRev4 primers were selected because they can also be used for the PCR Amplification of Specific Alleles (PASA) assay designed to detect the L1014F substitution described in previous work [13]. In this way, the presence of both mutations can be tested using the same primer set, with practical advantages for the parallel application of both diagnostic tools to field populations. PCR parameters were similar to those described above. The PCR products were further digested with SacI (Fermentas, Maryland, USA) according to the manufacturer's instructions. The products of digestions were analyzed in an agarose gel 2.5% stained with SybrGold (Invitrogen, São Paulo, Brazil). The protocol was tested with samples of known sequence (five replicas were performed).

## Results

**Figure 1:**
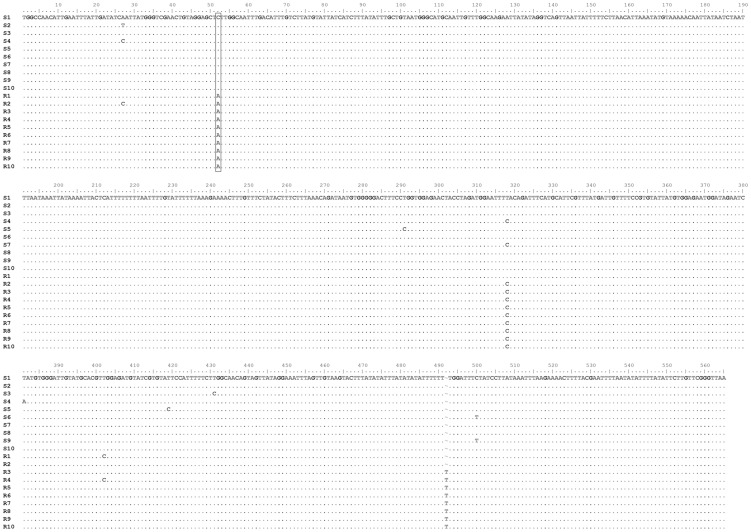
Alignment of the nucleotide sequences of a fragment from the voltage-sensitive sodium channel of *T. infestans*. Sequence of clones from sensitive (S) and resistant (R) populations are shown. Bases identical to those of the S1 clone are indicated by periods. A single non-silent substitution is detected in the position 52 of the sequence (signaled with the box), for every individual clone of R population.

**Figure 2:**
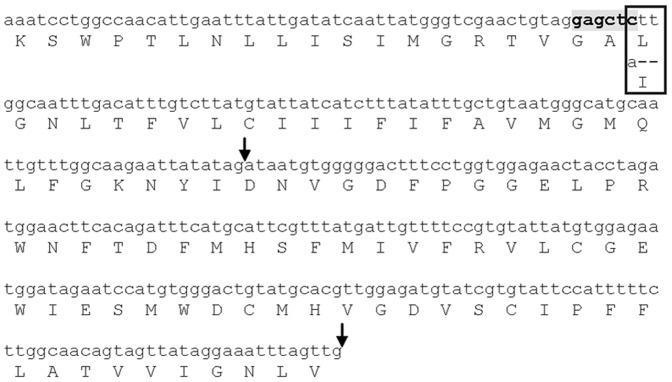
Nucleotide and predicted amino acid sequence of the portion of domain II of the *T. infestans para-*type sodium channel analyzed. The position of the L925I mutation is boxed. Vertical arrows indicate intron sites. Shadow indicates SacI restriction site.

Sequence analysis of the S and R insects reveals that all the sequenced clones from the S strain displayed the wild type sequence (GenBank accession number KF179338) (Figure 1). On the contrary, in all individual clones corresponding to the El Malá population, a single nonsilent nucleotide substitution is present (GenBank accession number KF179339). This substitution of adenine for cytosine produces a leucine to isoleucine mutation in the amino acid sequence (Figure 2). This mutation is located at an intracellular loop of the channel, between transmembrane segments IIS4 and IIS5 [12]. Furthermore, two additional silent base pair changes are strongly associated with the resistant strain (Figure 1); a substitution from thymidine to cytosine at base pair 318 (both codons encoding the amino acid phenylalanine), and an insertion of a thymidine at base pair 492 (inside the intron sequence).

**Figure 3:**
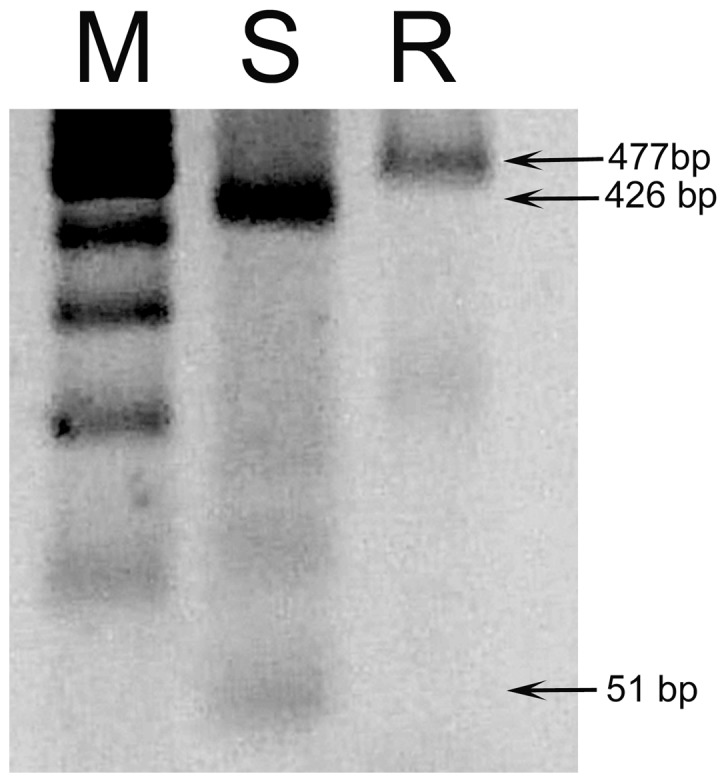
Assay for the detection of L925I mutation in individual bugs. The mutation suppresses a cleavage site for Sac I restriction endonuclease. As a result, when the susceptible allele (S) is present, two fragments are observed (51 bp and 426 bp). When the resistant allele is present (R), a single fragment of 477 bp is observed. M indicates the position of the standard size marker, in which the lower five bands are 100, 200, 300, 400 and 500 bp.

With the objective of developing a rapid assay to detect the L925I mutation in individuals from the field, we designed a method based on a different pattern of digestion by the restriction enzyme SacI; the nucleotide substitution of cytosine to adenine disrupts a restriction site for this enzyme. Figure 3 shows the results of a typical assay for samples of known genotype. After digestion, samples from susceptible individuals present one fragment of 426 bp and a second fragment of 51 bp; meanwhile, samples from resistant individuals, present a single fragment of 477 bp.

## Discussion

At the beginning of the SCI, the elimination of *T. infestans* was considered feasible. Evidence available at that time indicated a high susceptibility to pyrethroids and suggested little genetic variability, which would reduce the probability for insecticide resistance to emerge [2]. The latter assumption was later contradicted in other publications; *T. infestans* showed rich genetic variability through its distribution range [19]–[21]. Moreover, insecticide resistance was reported in several areas [5]–[10]. Today, insecticide resistance seems to be one of the principal reasons for the failure to eliminate *T. infestans* in the Gran Chaco ecoregion, even in areas subjected to intensive vector control efforts. Thus, resistance surveillance becomes a necessity for any Chagas control initiative in this region.

In this study, we report the *kdr* mutation equivalent to L925I in a highly resistant population of *T. Infestans* from the Gran Chaco. Along with our previous findings [13], these results implicate mutations in the sodium channel in pyrethroid resistance in *T. infestans*. The reduced susceptibility to deltamethirn of the insects from El Malá could reflect the fixation of a resistant-associated allele as a consequence of selection pressure with pyrethroids. However, considering the historical discontinuity in the chemical treatments and the high frequency of the resistant allele, L925I could represent the wild genotype of the insects from that area.

L925I has been previously found in pyrethroid resistant populations only in hemipteran species: the silverleaf whitefly *Bemisia tabaci* [14], the common bed bug *Cimex lectularius* [15], and the greenhouse whitefly *Trialeurodes vaporariorum* [16]. In the three species, populations carrying L925I mutation present levels of resistance >100, as is the case in *T. infestans*. L925I mutation has been functionally characterized in *X. laevis* oocytes, significantly decreasing pyrethroid potency [17]. L925 residue has been implicated as one of four pyrethroid binding residues in a model of the house fly sodium channel [22]. It is interesting to note that resistance levels in populations carrying the L925I mutation (RR = 1031) [10] are much higher than the ones observed in populations carrying L104F mutation (RR = 35.7) [13].

The identification of resistant-associated alleles in *T. infestans* represents an important advance in vector control, as it would allow the diagnosis of *kdr* alleles in the field, even if they are present in very low frequency. Here we present an assay based on different patterns of digestion by the restriction endonuclease SacI. This assay will allow the detection of L925I resistant mutation in insects from field populations, even in heterozygous individuals.

The assay presented here, along with the one described previously [13], toxicological bioassays and biochemical evaluations (i.e., activity of detoxifying enzymes), will provide a complete description of the resistance profiles of the different populations of *T. infestans*. The existence of other resistance-associated alleles cannot be ruled out and should be investigated. Such a diagnostic screening of populations will play an important role in adopting strategies for the management of resistance and in designing rational campaigns for the control of the insect vector of Chagas' disease. Specifically, the description of resistance profiles, including the resistance mechanisms involved, will allow the choice of the specific alternative insecticides for each population.
